# Direct On-Chip Differentiation of Intestinal Tubules from Induced Pluripotent Stem Cells

**DOI:** 10.3390/ijms21144964

**Published:** 2020-07-14

**Authors:** Elena Naumovska, Germaine Aalderink, Christian Wong Valencia, Kinga Kosim, Arnaud Nicolas, Stephen Brown, Paul Vulto, Kai S. Erdmann, Dorota Kurek

**Affiliations:** 1 Mimetas BV, Model Development, J.H. Oortweg 16, 2333 CH Leiden, The Netherlands; lnnaumovska@gmail.com (E.N.); aalderink.germaine@gmail.com (G.A.); k.kosim@mimetas.com (K.K.); a.nicolas@mimetas.com (A.N.); p.vulto@mimetas.com (P.V.); 2 Department of Biomedical Sciences, University of Sheffield, Western Bank, Sheffield S10 2TN, UK; cewongvalencia1@sheffield.ac.uk (C.W.V.); stephen.brown@sheffield.ac.uk (S.B.)

**Keywords:** iPSC, intestinal organoids, directed differentiation, gut-on-a-chip, organ-on-a-chip, microfluidics, 3D cell culture, intestinal inflammation

## Abstract

Intestinal organoids have emerged as the new paradigm for modelling the healthy and diseased intestine with patient-relevant properties. In this study, we show directed differentiation of induced pluripotent stem cells towards intestinal-like phenotype within a microfluidic device. iPSCs are cultured against a gel in microfluidic chips of the OrganoPlate, in which they undergo stepwise differentiation. Cells form a tubular structure, lose their stem cell markers and start expressing mature intestinal markers, including markers for Paneth cells, enterocytes and neuroendocrine cells. Tubes develop barrier properties as confirmed by transepithelial electrical resistance (TEER). Lastly, we show that tubules respond to pro-inflammatory cytokine triggers. The whole procedure for differentiation lasts 14 days, making it an efficient process to make patient-specific organoid tubules. We anticipate the usage of the platform for disease modelling and drug candidate screening.

## 1. Introduction

In-vitro models that closely mimic the human intestinal epithelium and its physiology are important tools for drug development processes [1,2,3]. As oral drug delivery is the preferred route of administration [4], such models have enormous potential to improve and speed up studies of absorption [5], disease modelling [6], drug metabolism and interactions as well as associated toxicity [7,8]. Therefore, patient-specific absorption models that take the gut complexity, passive and active transport and patient variability into consideration could be highly valuable in the evaluation of candidate drugs [9]. In addition to this, there is a strong need for patient-specific relevant models to mimic intestinal diseases such as inflammatory bowel disease and irritable bowel syndrome.

One potential cell source for building such models are intestinal organoids derived from induced pluripotent stem cells (iPSC) [10]. As opposed to human embryonic and adult stem cells, these cells are not associated with ethical concerns, they are easier to acquire and their generation does not involve the use of embryonic material [11]. Moreover, the ability of induced pluripotent stem cells to differentiate into the major cell types of the intestinal epithelium [5,12,13] makes them a useful tool to investigate the intestinal epithelium’s role in health and disease in-vitro. However, one of the major limitations of the organoid culture is the overall closed conformation, which makes the apical-luminal surface of the epithelium inaccessible [12,14] and, therefore, limits the applicability of these cultures for drug transport and absorption studies. Additionally, organoids are usually difficult and costly to produce, requiring one to two months to generate from the iPSC [15,16].

Several protocols exist for the directed in-vitro differentiation of iPSCs into intestinal tissue [12,17]. They usually follow a step wise addition of growth factors or small molecules to mimic intestinal morphogenesis and cytodifferentiation. In the first step definitive endoderm (DE) is usually acquired by applying an activin or Wnt-based protocol [18,19]. In the second step the culture is directed towards hindgut specification through FGF/Wnt induced posterior endoderm patterning [20,21,22]. In the last step, an intestinal like phenotype is generated by applying a cocktail of niche factors, including R-spondin, EGF, and the BMP inhibitor noggin [12,17,23]. These protocols generate human intestinal organoids (HIOs) containing both epithelial and mesenchymal cells. [17,24] Incorporation of these cells into microfluidics organ on a chip platforms (OOC) has the unparalleled potential of accelerating the development of medicines [25,26]. OOC techniques aid physiological relevance of cell cultures by adding perfusion flow, gradients, mechanical strain and controlled cell–cell interaction in a 3D environment [27,28,29]. Moreover, microfluidic approaches allow for both apical and basal access to the organoids. This feature is critical for many applications and the absence thereof is withholding routine adoption of current organoid techniques. However, incorporation of HIOs into microfluidic platforms usually requires an additional FACS-sorting step selecting the epithelial cell population based on the epithelial adhesion molecule (EpCAM) [17,24]. This additional step further increases the cost and time needed for incorporation of these cells in large-scale screening applications

In this study we report a protocol for directed differentiation of iPSC into 3D gut-like tubules directly in microfluidic devices. We make use of the OrganoPlate platform, which is a microtiter plate-based system, comprising 40 microfluidic chips. Chips comprise a freestanding extracellular matrix against which the tubule is grown, thus allowing for the interrogation of the epithelial barrier without interference of artificial membranes. We show that the cells follow a step wise differentiation through definitive endoderm, hindgut and the intestinal stage. They develop barrier function and apical to basal polarity over the course of two weeks. Additionally, tubules lose the stem cell markers and start expressing adult intestinal markers like efflux transporters P-glycoprotein (P-gp/MDR1) and drug-metabolizing enzymes cytochrome P450 3A4 (CYP3A4). We expose them to inflammatory cytokines in order to investigate the influence on barrier function and excretion of inflammatory cytokines. It is the first time that we show establishment of iPSC-derived tubules in microfluidic devices without the need for an intermediary organoid step. This method enables the quick and resource-efficient establishment of intestinal models from patient-specific cells that will prove crucial for routine adoption of microfluidic grown intestinal tubules for drug research and personalised medicine approaches.

## 2. Results

### 2.1. Directed on Organoplate Differentiation of iPSC Towards Gut-on-a-Chip

**Figure 1:**
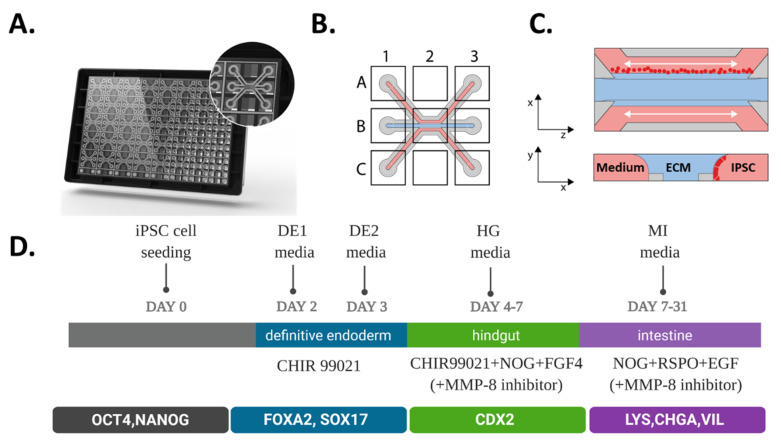
Seeding and differentiation of hiPSC in the 3-lane OrganoPlate. (**A**) Artist’s impression of the bottom-side of the 3-lane OrganoPlate containing 40 individual microfluidic chips. The inlay shows an individual chip that is communicating with nine connecting wells. (**B**) Scheme of a single chip containing three microfluidic channels all having top, middle and bottom inlets (A1, B1 and C1), outlets (A3, B3 and C3) and an observation window (B2). (**C**) A schematic representation of hiPSC introduction to a microfluidic chip in a top (xz plane) and cross sectional view (yz plane); single iPSCs (in red) are seeded in the top channel and are allowed to adhere for up to 24 h after which media is added and perfusion initiated, cells start to form tubules by first growing against the ECM gel (blue) and then covering the whole channel. (**D**) Schematic of workflow for directed on plate differentiation of iPSC towards gut tubules.

In this study, we made use of the 3-lane OrganoPlate platform which contains 40 independent microfluidic chips (Figure 1A) that have 3 microfluidic channels, each with dedicated inlets and outlets that join in the center of the chip. An observation window in the center of each chip allows microscopic observation of the intestinal culture (Figure 1B). An extracellular matrix (ECM) gel precursor is introduced into the middle channel where it is patterned by means of a surface tension technique called phaseguiding [30]. This yields a free-standing liquid–air meniscus in space of the gel precursor that is then allowed to solidify. Subsequently, iPSCs are seeded against the ECM gel in the top channel (Figure 1C). The experimental scheme is shown in (Figure 1D). Cells were directly seeded as stem cells in OrganoPlate, and after 2 days differentiation was started following the sequence of 2 days in Definitive endoderm (DE) media, 3 days of Hindgut (HG) media, and 7 to 24 days in the Mature Intestine (MI) media.

**Figure 2:**
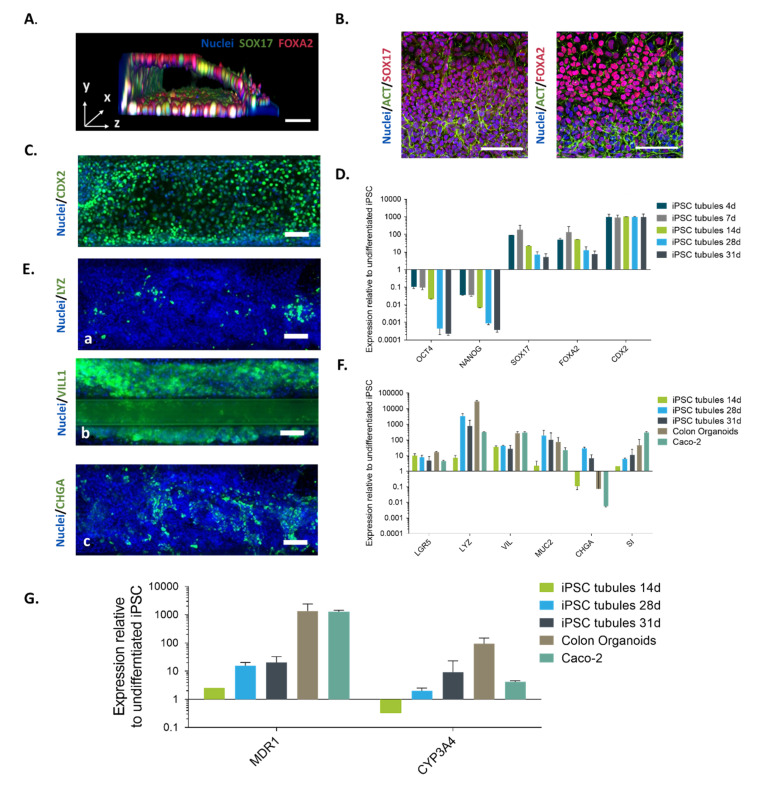
Characterization of hiPSC-derived human intestinal-like tubules. (**A**) 3D reconstruction image of an iPSC-derived tubule at Day 4 stained with antibodies for SOX17 (green), FOXA2 (red) or DAPI for DNA (blue). Cells attach and form tubule at the DE stage. (**B**) Representative images of iPSC tubules stained for Definitive endoderm markers SOX17 and FOXA2 at day 4. (**C**) Hindgut marker CDX2 at day 7. (**D**) Gene expression was measured using TaqMan qRT-PCR from hiPSC tubules at different differentiation stages. The following genes were analysed: POU class 5 homeobox 1 (POU5F1, indicating pluripotency); Nanog homeobox (NANOG, indicating Primitive Streak):, forkhead box a2 (FOXA2, Definitive Endoderm), SRY (sex determining region Y)-box 17 (SOX17, Definitive Endoderm), and Homeobox protein CDX-2 (Posterior gut). (**E**) Representative images of iPSC tubules stained for intestinal markers Lysozyme (LYZ), Villin (VIL) and Chromogranin A (CHGA) at day 28 (green). Nuclei were stained with DAPI (blue) to visualize the overall morphology. Scale bars = 100 µm. (**F**) Relative gene expression analysis of Intestinal markers: Leucine-rich repeat-containing G-protein coupled receptor 5 (LGR5), Mucin-2 (MUC2), Lysozyme (LYZ), Villin-1 (VIL1), Chromagranin A (CHGA) and sucrase-isomaltase (SI). (**G**) Relative mRNA expression of MDR1 (P-gp) and CYP3A4 in iPSC-derived intestine-like tubules on day 14, day 28 and day 31, human adult colon organoids and Caco-2 cells. Expression levels were normalized to beta-actin (ACTB), data represented as mean ± SD relative to the expression in undifferentiated miFF1 hiPSC (*n* = 2, n ≥ 2–3). The Y-axis represents the LOG10 relative quantification (RQ). Caco-2 cells and primary colon organoid were also included to compare gene expression and to follow the differentiation of our model during the different stages. Data are presented as the average of two independent experiments +/− SD (*n* = 3).

To optimize the attachment of iPSCs in the OrganoPlate, several conditions were tested including ECM composition (Collagen I and Matrigel) and coating strategies with recombinant human Vitronectin (rhVTN), Laminin and no coating at all (Figure S1). The stem cells attached to all coatings but detached from Laminin on day 4 and did not attach properly in the chips without coating. Moreover, conditions containing Matrigel as ECM and coated with Laminin or VTN were more likely to be subject to remodeling of the ECM by the cells, whereas chips having Collagen I and rhVTN as a coating remained more stable. Once we established and optimized the seeding strategy, we applied a 3-step directed differentiation protocol adapted for differentiation in microfluidic devices (Figure 1D). In the first step, the definitive endoderm (DE) differentiation is achieved by treating the iPSC for 24h with Wnt-agonist CHIR99021, followed by a medium only treatment. Moreover, to assess the differentiation potential of iPSC towards DE, we performed a small endodermal screen as described by Siler et al. (Figure S2A). iPSC were treated with either 3 or 4 μM CHIR99021 in RPMI/B27 +/− insulin in RPMI-1640 Medium on day 2 and RPMI/B27 +/− insulin in RPMI-1640 on day 3. We did not observe any morphological differences between the treatments since cells were able to form tubules in all conditions (Figure 2A). There were no significant changes in DE and HG marker expression as tested by RT-qPCR. TEER measurements revealed minimal, but not significant barrier function in the 3 μM CHIR99021 in RPMI/B27 + insulin in RPMI-1640 medium condition. The uniform expression of FOXA2 and SOX17 endodermal markers on day 4 confirms successful differentiation towards the definitive endoderm (Figure 2B).

Activation and inhibition of several signaling pathways like Notch, Wnt, phospoinositide-3-kinase and BMP are important for the intestinal differentiation [31]. Therefore, in the second step we examined whether DE cells can be differentiated towards the hindgut (HG) stage within the microfluidic platform. Cells were exposed to CHIR 99020, BMP-antagonist Noggin (NOG) and Fibroblast growth factor 4 (FGF4) for 3 days. Figure 2A shows a 3D representation of a tubule at day 4. Figure 2A,B show expression of markers SOX17 and FOXA2 that are representative of the definitive endoderm stage. In standard culture, the iPSC-derived intestinal organoids form and detach from the cell culture surface at this stage [12]. Here we confirm that cells form and maintain a tubular shape. On day 7 post-seeding, cells started expressing the intestinal progenitor cell marker caudal type homeobox 2 (CDX2) transcription factor, which is an important factor for intestinal development [32,33] (Figure 2C). Moreover, cells showed downregulated expression of the pancreatic progenitor cell marker pancreatic and duodenal homeobox 1 (PDX1), confirming that the cells were of a posterior nature (Figure S2). This result suggests that iPS cells were successfully differentiated towards intestinal lineage.

In the last step of the differentiation protocol a mature intestinal media (MI) was added to direct the CDX2+ cells towards more mature intestinal cells. The medium was enriched with epidermal growth factor (EGF), Wnt agonist R-spondin1 (Rspo1) and Noggin (NOG) emanating the signalling molecules from supporting mesenchyme for stem cell homeostasis [34]. On day 14 (a week after starting with MI media), expression of mature intestinal markers was detected by qPCR and the expression was pronounced at day 28. Stem cell marker Leucine-rich repeat-containing G-protein coupled receptor 5 (LGR5), goblet cell marker Mucin-2 (MUC2) and brush border sucrase-isomaltase (SI) were readily detected and on levels similar to human adult colon organoids and Caco-2 (Figure 2F). Additionally, we detected expression of the Paneth cell marker Lysozyme (LYZ), the enterocyte cell marker Villin-1 (VIL1) and the neuroendocrine cell marker Chromogranin A (CHGA) which were distributed throughout the tubule (Figure 2E).

Next, we tested the potential suitability of our model for drug studies. Therefore, we first examined the gene expression levels of the drug metabolizing enzymes cytochrome P450 3A4 (CYP3A4) and multidrug resistance protein 1 (MDR1) by real-time RT-PCR analysis. These molecules are important in drug development, because they are involved in reducing the drug absorption and limiting the systemic bioavailability [35,36,37,38]. The gene expression levels of both CYP3A4 and MDR1 in the hiPSC-derived tubules were lower than those in adult human colon organoids. However, when compared to cells with low expression like Caco-2 cells [39] the hiPSC-derived tubules had 4 times higher expression levels of CYP3A4 (Figure 2G).

One of the major obstacles during the development of this model was the invasion of cells into the Collagen I ECM which usually happened at the second step or the HG stage, approximately 9 days post-seeding in the chip. While invasion and remodelling of the ECM are normal processes in the epithelium of the gut, it affected barrier function as became apparent in the TEER measurements. Initially, we attempted to apply cost-effective solutions where we increased the concentration of ECM in order to strengthen the gel and reduce the movement of the gel; however, with limited success. Therefore, we looked into preventing ECM breakdown by inhibition of matrix metalloproteinases (MMPs). We tested both a MMP-8 specific inhibitor (CAS 236403-25-1) and a broad spectrum MMP-inhibitor Marimastat (CAS 154039-60-8) at a 10-µM concentration [40] added from day 4 onwards. The addition of MMP-8 inhibitor prevented the invasion of cells into the ECM and helped in maintaining tubule integrity (Figure S3A) while it did not affect the expression of mature intestinal markers (Figure S3B). Broad spectrum MMP-inhibitor (Marimastat), even though effectively preventing ECM invasion, did affect cell morphology (Figure S3A).

### 2.2. iPSC-derived Gut-Like Tubules Develop Barrier Properties

**Figure 3:**
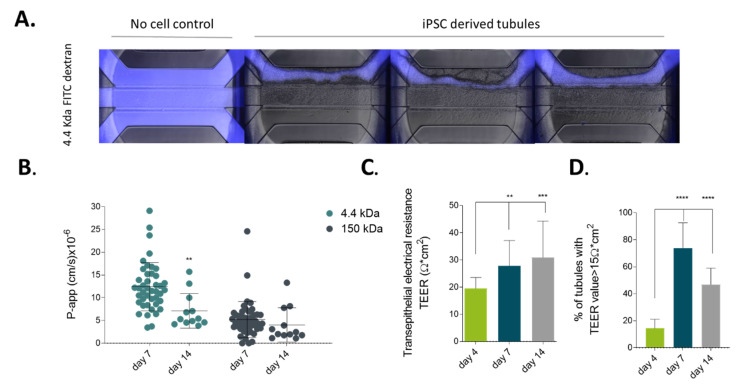
Barrier function of iPSC-derived intestinal-like tubules. (**A**) Merge of fluorescent and phase contrast images of four microfluidic chips perfused in the top channel with fluorescent molecules (in blue) using 4.4 kDa TRITC-Dextran for 15 min on day 25 of culture. The ‘no cell control’ shows the passage of the fluorescent dye into the adjacent gel and secondary perfusion channel, whereas in the chips with iPSC tubules the fluorescent dye is retained by the tubule. (**B**) P-app value calculated based on a fluorescent permeability assay using both 4.4 kDa TRITC-dextran and 150 kDa FITC-dextran at day 7 and day 14. Data are represented as mean ± SD. Significance was detected by two-way Anova, n ≥ 12. (**C**) Transepithelial electrical resistance (TEER) measurements of iPSC-derived intestine-like tubules between day 4 and day 14 of culture (N = 3, n ≥ 110). (**D**) Percentage of iPSC-derived intestine-like tubules between day 4 and day 14 of culture developing TEER values above 15 Ω cm^2^. Significance was detected by ordinary one-way Anova with Dunett’s multiple comparison test. Data are represented as mean ± SD. (*n* = 3, *n* ≥ 110), ns *p* > 0.05, * *p* ≤ 0.05, ** *p* ≤ 0.01, *** *p* ≤ 0.001, **** *p* ≤ 0.0001.

To determine whether the hiPSC-derived tubules could be applicable to drug permeability studies, we evaluated barrier function of the tubules by performing a real-time permeability assay and transepithelial electrical resistance (TEER) measurements as previously described by Trietsch et al. and Beaurivage et al. [6,41]. Figure 3A shows the barrier function of iPSC-derived intestinal tubules. In short, tubules were perfused with a fluorescently labeled dextran (Figure 3A in blue). In the case of a tubule, the dye was contained to the lumen, whereas for the ‘no cell control’, fluorescence was observed all through the microfluidic structure, including adjacent gel layer and secondary perfusion channel. This demonstrates that tubules form leak-tight barrier. To further assess barrier function, we calculated the apparent permeability of the tubes over time (see Figure 2B). As shown, tubules became more leak-tight over time and appeared to be most leak-tight on day 14, with a P-app score of 4.03 × 10^−6^ cm/s for 150 kDa Dextran and 7.12 × 10^−6^ cm/s for 4.4 kDa. The TEER value is representative for the integrity of tight junction dynamics in cell culture models of endothelial and epithelial monolayers. Tubules were subjected to TEER measurement after confluency was reached on day 4 and up till day 14 (Figure 3C). TEER values reached a plateau of 30.91 ± 13.37 Ω cm^2^ on day 14 and maintained this plateau at least until day 21 (data not shown). Interestingly, approximately 90% of tubules developed a barrier starting from day 7 (Figure 3D) and after 2 weeks of differentiation these iPSC-derived gut-like tubules exhibited TEER values similar to primary intestinal tissue with values between 40–100 Ω cm^2^ [42].

### 2.3. Modelling Infammation in iPSC-derived Tubules

**Figure 4:**
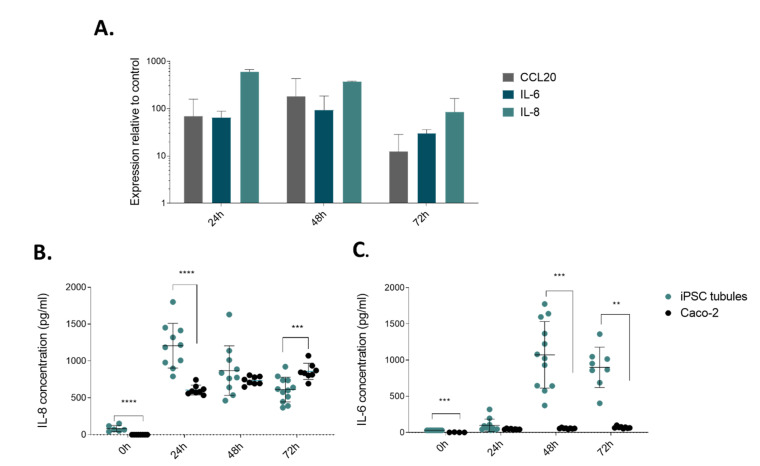
hiPSC-derived gut tubules respond to inflammatory triggers. (**A**) Gene expression analysis of hiPSC-derived gut tubules for CCL20, IL-8, and IL-6 upon stimulation with cytokine cocktail of TNF-α, IL-1B and IFN-γ. Expression levels are measured upon 24 h, 48 h and 72 h of exposure to the cytokine cocktail. Data are represented as mean ± SD normalized to ACTB expression (*n* = 2–3). (**B**) Secretion of IL-8 and (**C**) secretion of IL-6 by triggered hiPSC-derived gut tubules (blue) and Caco-2 tubules (gray) upon stimulation with the cytokine cocktail. Significance was determined with Multiple t-tests (one per row) and discovery determined using the Two-stage linear step-up procedure of Benjamini, Krieger and Yekutieli, with Q = 1%. Each row was analyzed individually, without assuming a consistent SD. Data are represented as mean ± SD. (*n* ≥ 5) ns *p* > 0.05, * *p* ≤ 0.05, ** *p* ≤ 0.01, *** *p* ≤ 0.001, **** *p* ≤ 0.0001.

We assessed applicability of our model for disease modeling of inflammatory responses as occur in patients with inflammatory bowel disease. We applied an inflammatory trigger composed of TNF-α, IL-1B and IFN-γ [6,43]. These cytokines are known to be upregulated in several inflammatory conditions. We focused on specific aspects of intestinal inflammation like expression and secretion of pro-inflammatory cytokines by activated epithelial cells. CCL20 is a chemo attractant to recruit circulating Th17 cells to sites of inflammation [44], IL-6 is a cytokine with main function to regulate T cell balance [45], and IL-8 is a powerful neutrophil chemoattractant found in inflamed mucosa [46]. Once added to the tubules, the cytokine cocktail upregulated mRNA expression and the maximal expression was observed after 48 h for CCL20 and IL6, and the highest expression levels for IL-8 were observed at 24 h (Figure 4A).

We next investigated the release of IL-6 and IL-8 by iPSC gut tubules compared to Caco-2 tubules, in response to cytokine cocktail exposure, using enzyme-linked immunosorbent assay (ELISA). IL-8 production after the addition of the inflammatory trigger to the iPSC-derived intestinal tubules was significantly increased after the first 24 h, and then decreased significantly for each of the following 24 h, decreasing from a mean of 1206 ± 303 pg/mL to 869 ± 336 pg/mL at 48 h and 613 ± 167 pg/mL at 72 h. The IL-8 secretion of Caco-2 tubules showed a reversed tendency, increasing from 607 ± 66 pg/mL at 24 h, to 734 ± 55 pg/mL at 48 h and 859 ± 109 pg/mL at 72 h (Figure 4B).

After cytokine stimulation, the IL-6 production was high for the iPSC-derived tubules 97.40 ± 86.5 pg/mL, 1072 ± 460 pg/mL and 898 ± 278 pg/mL at 24 h, 48 h and 7 2h, respectively. Caco-2 tubules had low IL-6 production at all of the time points (Figure 4C). We thus conclude that iPSC-derived tubules are more responsive to cytokine stimuli than Caco-2 tubules, indicating a more physiologically relevant behavior.

## 3. Discussion

In summary, we developed a fully defined and partially small molecule-based differentiation protocol [10,12,24] to directly differentiate from iPSC towards gut-like tubules in a microfluidic platform. Tubules were successfully differentiated to definitive endoderm after 4 days in culture and reached expressed hindgut markers at day 7. We showed the upregulation of MDR1 and CYP3A4—enzymes involved in intestinal drug metabolism. Furthermore, the intestinal tubules were shown to have a barrier function as assessed by both barrier integrity assay and TEER measurements. We demonstrated that these tubules respond to inflammatory cytokines. Other than Caco-2 cells, our intestinal tubes show increased production of IL-6 upon stimulation. We thus anticipate that the model can be used for ADME tox studies as well as for modelling intestinal inflammation.

The main purpose of this study was to develop a method to generate iPSC-derived gut-like tubules directly, robustly and reproducibly in a microfluidic platform, while omitting the long and labor-intensive intermediate step of organoid generation. Thus, we reduce culture and differentiation times, as well as usage of media and associated growth factors. This is of crucial importance, to consider microfluidics-based culture for routine use in drug development and basic research alike. An advantage of using such microfluidic techniques is that we have straightforward access to both the apical as well as basal side of the epithelium.

For the differentiation of the iPSC cells towards definitive endoderm in the OrganoPlate, we used a short pulse of small molecule Wnt activator CHIR99021 instead of the recombinant Activin A molecule [17] based protocol. GSK-3 inhibition by CHIR99021 alone is sufficient for the generation of SOX17 and FOXA2 positive cells [19,47]. This step shortens the definitive endoderm phase from 5 days to 2 days. In the subsequent step FGF4 and CHIR99021 signaling were applied as they are known to induce the expression of the posterior endoderm marker *CDX2* [12,48]. This stage of differentiation can also be achieved by applying a BIO/DAPT combination of small molecules [49]. However, BIO is known to have various off target effects including inhibition of the JAK/STAT signals [50], which are necessary for intestinal proliferation [51]. We further demonstrated that a combination of niche factors including Noggin, R-spondin and EGF are sufficient to induce the differentiation of the iPSCs towards intestinal progenitor cells. Recombinant protein (e.g., Wnt3a) nor a small molecule Wnt agonist (e.g., CHIR99021) are not used in this step. However, since the differentiated epithelium includes Paneth cells, we assume that the stem cell niche is supported [52]. In future studies, we will further investigate this aspect.

We showed the applicability of our model for permeability studies by performing large scale TEER measurements (*n* = 110) over 14 days of culture in the microfluidic device. Thus, demonstrating that our model can develop a barrier starting from day 4 and reaching levels similar to the primary tissue in 14 days between 40–100 Ω cm^2^ [42] with the addition of a MMP-8 inhibitor. In contrast, the average TEER values for Caco-2 cultures ranges between 500–1500 Ωcm^2^ [53,54]. For the applicability of the model in drug discovery studies, it should be noted that the P-gp transporter (MDR1) expression is lower than for the human colon Organoids and Caco-2 tubules. However, this is consistent with previous findings [49,55,56] of stem cell differentiated gut-like tissue, and suggests that our hiPSC-derived gut-like tubules resemble the fetal tissue rather than the adult one.

Lastly, we were able to demonstrate that our system is susceptible to stimulation with inflammatory cytokines, demonstrating the potential for modeling an inflammatory condition such as is present in patients with inflammatory bowel disease. The iPSC-derived intestinal-like tubules showed a significant increase in IL-6 and IL-8 release after 48 and 24 h, respectively. While Caco-2 cells are capable of producing IL-8 after several days of culture [57], in the literature there is conflicting evidence regarding IL-6 secretion by Caco-2 [17,58]. In our experiments, we were not able to detect it. The function of interleukin-6 in the intestinal epithelium is to increase proliferation and survival by an autocrine signaling mechanism [59]. Cells that express IL-6 receptors are crypt Paneth cells [43,60]. Caco-2 cells, when differentiated, are known to resemble enterocytes of the small intestine and, therefore, it is explainable that they are unable to produce IL-6 in large quantities [61].

In conclusion, in this proof-of-principle study, we demonstrated the possibility for iPSCs to be successfully directed towards gut-like phenotype in a robust, reproducible and cost-effective manner, all in a 3D microfluidic device and within 14 days post-seeding. Moreover, the iPSC develops into relevant leak-tight tubules with the expression of intestinal markers, similar to existing models, and with significant response to inflammatory triggers. This allows for the efficient establishment of patient-specific in-vitro models, which have the potential to be used for drug discovery and for studying inflammatory processes in the intestine.

## 4. Materials and Methods

### 4.1. Ethics Statement

The research described here has been performed according to applicable Dutch and UK national ethics regulations and was conducted within MIMETAS BV (Leiden, The Netherlands) and the University of Sheffield (Sheffield, United Kingdom). Colon organoid line was previously derived at the Hubrecht institute, Utrecht, the Netherlands from colonic tissue obtained from The Diakonessen Hospital Utrecht with informed consent and the study was approved by the ethical committee [62].

### 4.2. Cells

Human induced pluripotent stem cells (hiPSC; miFF1 UOSi001-A) [63] were obtained from the University of Sheffield (Sheffield, United Kingdom), cultured in a standard gas atmosphere with 95% humidity and 5% CO_2_ at 37 °C in feeder-free conditions using 0.5 mg/mL Vitronectin Recombinant Human Protein (Thermo Fisher Scientific No. A14700)-coated 6 well plates. Routine passaging of cells was performed with 0.5 mM EDTA (Thermo Fisher Scientific, 15-575-020). Essential 8 medium (Thermo Fisher Scientific No. A1517001) supplemented with 10 µM Y-27623 ROCK inhibitor (Merck No. 688000-1MG) was used in the first 24 h post-passaging. Essential 8 medium (Thermo Fisher Scientific No. A1517001) was used for the expansion after the initial 24 h.

Human colon adenocarcinoma cell line Caco-2 (ECACC 86010202) was cultured as described earlier [6,41]. In short, Caco-2 cells were cultured at 5% CO_2_ at 37 °C in an EMEM (ATCC, Manassas, VA, USA) supplemented with 10% FBS (Gibco, Waltham, MA, USA), 1% NEAA (Gibco, Waltham, MA, USA) and 1% penicillin/streptomycin (Gibco, Waltham, MA, USA). Cells were passaged before reaching 80% confluency and used between passage 47–60.

Human Colon Organoids were obtained from the Hubrecht Institute (Utrecht, Netherlands) and cultured as described previously [62]. In short, organoids were embedded in Matrigel GFR (Corning, 356231) and cultured in 24-well plates in 1× CNM media containing 50% Wnt3a CM, Advanced Dulbecco’s modified Eagle medium/F12 (ThermoFisher 12634028) supplemented with penicillin/streptomycin, 10 mmol/L HEPES, 1× Glutamax, 1× B27 supplement, 1.25 mM *N*-acetyl-cysteine (Sigma A9165), 10 mM Nicotinamide (Sigma, N0636), 5 nM Gastrin (Sigma, G9145), 100 ng/mL Noggin (Peprotech 120-10C), 250 ng/mL Rspondin-3 (Tocris, 3500), 50 ng/mL EGF (Peprotech, AF-100-15), 500 nM A83.01 (Tocris 2939), 10 µM SB2020190 (Sigma, S7076) and 50 μg/mL Primocin (Invivogen ANT-PM-2). Culture was passaged every 7 days and Y-27632 dihydrochloride (Torcis 1254) was added to the media for the initial 48 h.

Cell were routinely tested against mycoplasma contamination and were found negative.

### 4.3. OrganoPlate Seeding and Tubule Formation

ECM loading was prepared as described previously in a 3-lane 400 µm OrganoPlate (Mimetas No. 4003-400-B) [41,64]. In short, 1.5–2 µL of 6.64 mg/mL collagen-I (Corning,354249) was dispensed into the middle inlet. The OrganoPlate was placed in the incubator for 15 min to allow the polymerization of the collagen-I gel. After solidification of the gel, 30 µL HBSS was placed in the middle inlet to prevent the gel from drying out and the plate was placed back into the incubator until use. Then the OrganoPlate was coated with 0.5 mg/mL Vitronectin Recombinant Human Protein (Thermo Fisher Scientific, A14700) diluted in ice-cold PBS added in the top medium inlet for 60 min at room temperature (RT) before seeding. Cells were dissociated to a single cell suspension and the concentration was adjusted to 5000 cells/µL in mTeSR medium supplemented with 10 µM Y-27623 ROCK inhibitor. HBSS was removed from the gel inlets and 2 µL cell suspension was inserted in the top medium outlet. Next, 1 µL of liquid was removed from the top medium inlet to assist the seeding of cells in a coated channel. After which, 50 µL of mTeSR medium (STEMCELL Technologies,85850) supplemented with 10 µM Y-27623 ROCK inhibitor was added to the top medium inlet to prevent evaporation. The OrganoPlate was placed flat and static in a humidified chamber to allow the cells attachment for 6 h. Subsequently, 50 µL of mTeSR medium was added to the top medium inlet, bottom medium inlet and outlet as well. Cells were then incubated in a humidified chamber on an interval rocking platform switching between a +7° and –7° inclination every 8 min (OrganoFlow; Mimetas BV), to induce bidirectional flow.

### 4.4. Directed on Plate Differentiation of iPSC

After the initial 48 h of mTeSR medium in the Organoplate, cells were exposed to definitive endoderm differentiation medium (DE) Table S1. After additional 48 h the differentiation on the definitive endoderm was completed, the cultures were started on Hindgut differentiation medium on day 4 (HG) Table S1. These conditions were maintained for 3 days. Cells were further directed towards the mature intestine with mature intestine medium (MI) Table S1 for an additional two to three weeks, with medium changes every 3 to 4 days (Table S1). An amount of 10 µM MMP-8 inhibtor (Fischer Scientific No. 44-423-7) or 10 µM Marimastat (MERCK,. M2699) was added to the cultures once the Hindgut stage of differentiation was started to reduce the invasion of cells into the ECM.

### 4.5. Barrier Intergrity Assay (BI Assay) and Calculation of Aperent Permeability (P-App)

The barrier integrity assay was performed to assess the barrier integrity of a boundary tissue and has been described in detail previously [6,41,64]. In short, basal media was added to the middle and bottom inlets and outlets. The top inlets or the lumens of the intestinal tubules were perfused with media containing high 0.5 mg/mL FITC-dextran (150 kDa, Sigma No. 46946) and low molecular weight TRITC-dextran (4.4 kDa, Sigma No. T1037). Plates were imaged using ImageXpress XLS Micro HCI system with an interval of every 3 min for total time of 15 min. The permeability of the fluorescent particles was quantified by determining the fluorescence levels in the basal gel region and normalizing it to the fluorescence in the lumen of the tubule to compensate for bleaching effects. The ratio between the fluorescent signal in the basal and apical region of the tube was analyzed using Fiji. Cell-free chips were taken as negative controls. These data were then used to calculate the apparent permeability value (P-app) according to van Duinen et al. [65].

### 4.6. TEER Measurements

Transepithelial electrical resistance (TEER) was measured at different time points using an automated multichannel impedance spectrometer designed for use with the OrganoPlate (OrganoTEER, Mimetas). Before each measurement, medium was added in the middle inlets and outlets and the OrganoPlate was returned in the incubator (37 °C, 5% CO_2_) to equilibrate for an hour. The electrode board of the OrganoTEER is matched to the OrganoPlate, such that when an OrganoPlate is placed in the OrganoTEER, electrode pairs are inserted in the medium in all inlet and outlet wells connecting to the basal and apical side of all tubes. Point impedance measurements were performed at room temperature by frequency sweep from 10 Hz to 1 MHz (41 points; precision, 0.2). Data were analyzed using the OrganoTEER software, which automatically extracts the TEER contribution (in Ohm) from the measured spectra and normalizes it to Ohm.cm^2^ by multiplying by the tubule-ECM interface (estimated at 0.0056 cm^2^).

### 4.7. Cytokine Trigger and ELISA for Activation Molecules

iPSC and Caco-2 tubules treated with cytokine-triggering medium consist of IL-1β, IFN-γ and TNF-α at 50, 50 and 50 ng/mL, respectively (ImmunoTools, Friesoythe, Germany), for 24, 48 and 72 h. Medium was collected from the Top and Bottom inlets and outlets, pooled and stored at −80 °C until further analysis. CXCL6 and CXCL8 secretion were quantified using the Human IL-6 DuoSet ELISA (RD systems No. DY206) and Human IL-8/CXCL8 DuoSet ELISA (RD systems No. DY208), respectively, according to the manufacturer’s instructions.

### 4.8. Immunocytochemistry (ICC)

Cells were fixated with 3.7% formaldehyde in HBSS at RT for 15 min. Cells were washed twice with PBS for 5 min and subsequently washed once with 4% Fetal bovine serum (FCS) in PBS for 5 min, followed by permeabilization with 0.3% Triton X-100 in PBS for 10 min. After which, the cells were washed with 4% Fetal bovine serum (FCS) in PBS for 5 min and blocked with 2 % FCS, 2% Bovine serum albumin (BSA), 0.1% Tween-20 in PBS for 30 min. Next, cells were incubated overnight with primary antibodies diluted in blocking buffer. Cells were washed with 4% Fetal bovine serum (FCS) in PBS twice for 3 min and subsequently incubated with secondary antibodies for 30 min, followed by a washing step and nuclei staining with Hoechst 33342. The antibodies used: SOX17 (R&D Systems, MAB19241 1/100), FOXA2 (R&D Systems AF2400. 1/100) CDX2 (R&D Systems, AF3665, 1/100), Villin (Santa Cruz, 58897, 1/200), Chromagranin A (Rabbit Santa, cruz13090, 1/200), Lysozyme (Thermo Fischer, MA1-82873,1/200), Isotype Rabbit (Life Technologies, 86199, 1/250) Isotype Goat (Life Technologies, 026202, 1/250), Isotype Mouse (Invitrogen, 08-6599 1/250), Goat Alexa Fluor 488 (Invitrogen, A11055 1/250) Mouse Alexa Fluor 555(Life Technologies, 1736967, 1/250), Rabbit Alexa Fluor 647 (MERCK, SAB460177 1/250) and cell stains Actin Green (LifeTechnologies, R37110), Hoechst 33342 (Thermo Fischer, H3570, 1/1000).

### 4.9. Gene Expression Anaysis

Total RNA was isolated using RNeasy Micro kit (Qiagen No. 74004) according to the manufacturer’s instructions; two to three chips were pooled into one sample. We first determined the RNA concentration with NanoDrop OneC Microvolume UV-Vis Spectrophotometer (Thermo Fischer) and adjusted it to a min concentration of 30 ng/μL in DEPC-treated milliQ water. M-MLV reverse transcriptase (28025013; Thermo Scientific) was used to synthesize cDNA by following the manufacturer’s protocol. Finally, qPCR was performed by using FastStart Essential DNA Green Master (Roche, 06402712001) for SYBR Green I-based real-time PCR and FastStart Essential DNA Probe Master (Roche, 06402682001) for TaqMan based real-time PCR.

SYBR Green primers used during experiments. GeneID, NCBI number, Primer sequence 5′–3′:

ACTB NM_001101.3

F: CTCTTCCAGCCTTCCTTCCT R: AGCACTGTGTTGGCGTACAG,

SI NM_001041.3

F: GTAAGGAGAAACCGGGAAGC R: TGTCCATGGTCATGCAAATC,

CYP3A4NM_001202855.3

F: TTTACCCAATAAGGCACCACC R: TTGCAGACCCTCTCAAGTC

CXCL6 NM_002993.4

F: TGTTTACGCGTTACGCTGAG R: AACTTGCTTCCCGTTCTTCA

CXCL8 NM_000584.4

F: CAAGAGCCAGGAAGAAACCA R: ACTCCTTGGCAAAACTGCAC

CCL20 NM_004591.3

F: GCAAGCAACTTTGACTGCTG R: GATGTCACAGCCTTCATTGG

TaqMan probes for: OCT4 Hs04260367_gH FAM 77 NANOG Hs04260366_g1 FAM 99 SOX17 Hs00751752_s1 FAM 149 FOXA2 Hs05036278_s1 FAM 144 CDX2 Hs01078080_m1 FAM 81 PDX1 Hs00236830_m1 FAM 73 LGR5 Hs00969422_m1 FAM 61 LYZ Hs00426232_m1 FAM 67 VIL1 Hs01031722_g1 FAM 72 MUC2 Hs00159374_m1 FAM 81 CHGA Hs00900375_m1 FAM 88 ABCB1 Hs00184491_m1 FAM 110 ACTB Hs01060665_g1 VIC 63 06402712001)

Results were normalized relative to ACTIN expression for both Taqman and SybrGreen experiments.

### 4.10. Statistics and Data Analysis

Data were analyzed using GraphPad Prism software version 7 (GraphPad Software, La Jolla, CA, USA). Unless stated otherwise, values are expressed as mean ± Standard Deviation (SD). ANOVA and Student’s t-tests were used to determine the statistical significance. Differences with *p* < 0.05 were considered significant (ns *p* > 0.05, * *p* < 0.05, ** *p* < 0.01, *** *p* < 0.001, **** *p* < 0.0001). All graphs shown contain results from one or two representative experiments containing at least three technical replicates (chips). Number of replicates used are provided in the figure legends.

## Supplementary Materials

The following are available online at https://www.mdpi.com/1422-0067/21/14/4964/s1, Figure S1: Optimization of ECM and coating strategy for better attachment and tubule maintenance of iPSC; Figure S2: Endodermal potential screen and barrier integrity of iPSC; Figure S3: Maintenance of tubular shape of differentiated iPSC derived gut-like tubules within a microfluidic device with matrix metalloproteinases inhibitors; Table S1: Components of directed differentiation media.

## Abbreviations

CD	Crohn’s disease
CYP	Cytochrome P450
DE	Definitive endoderm
EGF	Epidermal growth factor
ESCs	Embryonic stem cells
HG	Hindgut
HIOs	Human Intestinal Organoids
FGF4	Fibroblast growth factor 4
IBD	Inflammatory bowel disease
iPSC	induced pluripotent stem cells
LGR5	Leucine-rich-repeat-containing G-protein-coupled receptor 5
MI	Mature intestine
MMP	Matrix metalloproteinase
P-gp	P-glycoprotein
RNase	Ribonuclease
ROCK	Rho-associated protein kinase
TEER	Trans epithelial electrical resistance
