# Supplementary material for: Direct On-Chip Differentiation of Intestinal Tubules from Induced Pluripotent Stem Cells
Source: Int J Mol Sci. 2020 Jul 14;21(14):4964. doi: 10.3390/ijms21144964 (PMC7404294; doi:10.3390/ijms21144964)
Supplement: Supplementary file 1 [file ijms-21-04964-s001.pdf]

Table S1. Components of directed differentiation media

Stage of differentiation	Abbreviation	Component	Final concentration	Supplier	Cat.No
Definitive endoderm	DE1	RPMI-1640 Medium	/	Sigma-Aldrich	R0883
		Penicillin-Streptomycin	1x	Sigma-Aldrich	P4333
		Non-essential amino acids	1x	Gibco	11140035
		B27 with insulin	1x	Gibco	17504-044
		CHIR99021	3 uM	Bio-Techne	4423
	DE2	RPMI-1640 Medium	/	Sigma-Aldrich	R0883
		Penicillin-Streptomycin	1x	Sigma-Aldrich	P4333
		Non-essential amino acids	1x	Gibco	11140035
		B27 with insulin	1x	Gibco	17504-044
Hindgut	HG	RPMI-1640 Medium	/	Sigma-Aldrich	R0883
		Penicillin-Streptomycin	1x	Sigma-Aldrich	P4333
		Non-essential amino acids	1x	Gibco	11140035
		FBS	2%	ATCC	30-2020
		CHIR99021	2 uM	Bio-Techne	4423
		FGF 4	500ng/mL	Peprotech	100-31
		Noggin	100ng/mL	Peprotech	120-10C
Mature intestine	MI	DMEM/F12 Medium	/	Gibco	31331-028
		HEPES	15mM	Gibco	15630-056
		Penicillin-Streptomycin	1x	Sigma-Aldrich	P4333
		Non-essential amino acids	1x	Gibco	11140035
		B27 with insulin	1x	Gibco	17504-044
		Noggin	100 ng/mL	Peprotech	120-10C
		R-spondin	500 ng/mL	Peprotech	120-38
		EGF	100 ng/mL	Sigma-Aldrich	E9644-.2MG
		MMP-8	10 uM	Fisher Scientific	4442371MG

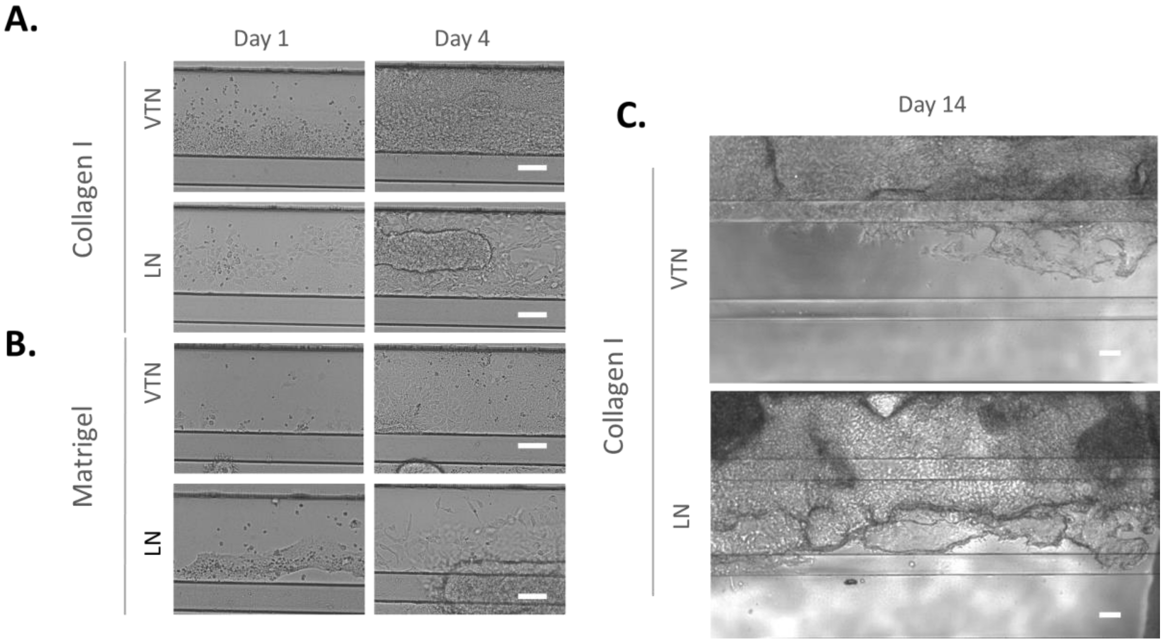

Figure S1. Optimization of ECM and coating strategy for better attachment and tubule maintenance of iPSC. Representative 10X phase contrast images of iPSC derived tubules at Day 1 and 4 cultured on Collagen I and human recombinant vitronectin (VTN) or laminin (LN) as coating strategy (A) or Matrigel as ECM and human recombinant vitronectin (VTN) or laminin (LN) as coating strategy (B). C. Representative 10X phase contrast images of iPSC derived tubules at Day 14 with Collagen I as ECM and VTN or LN as a coating strategy. Scale bars=100μm

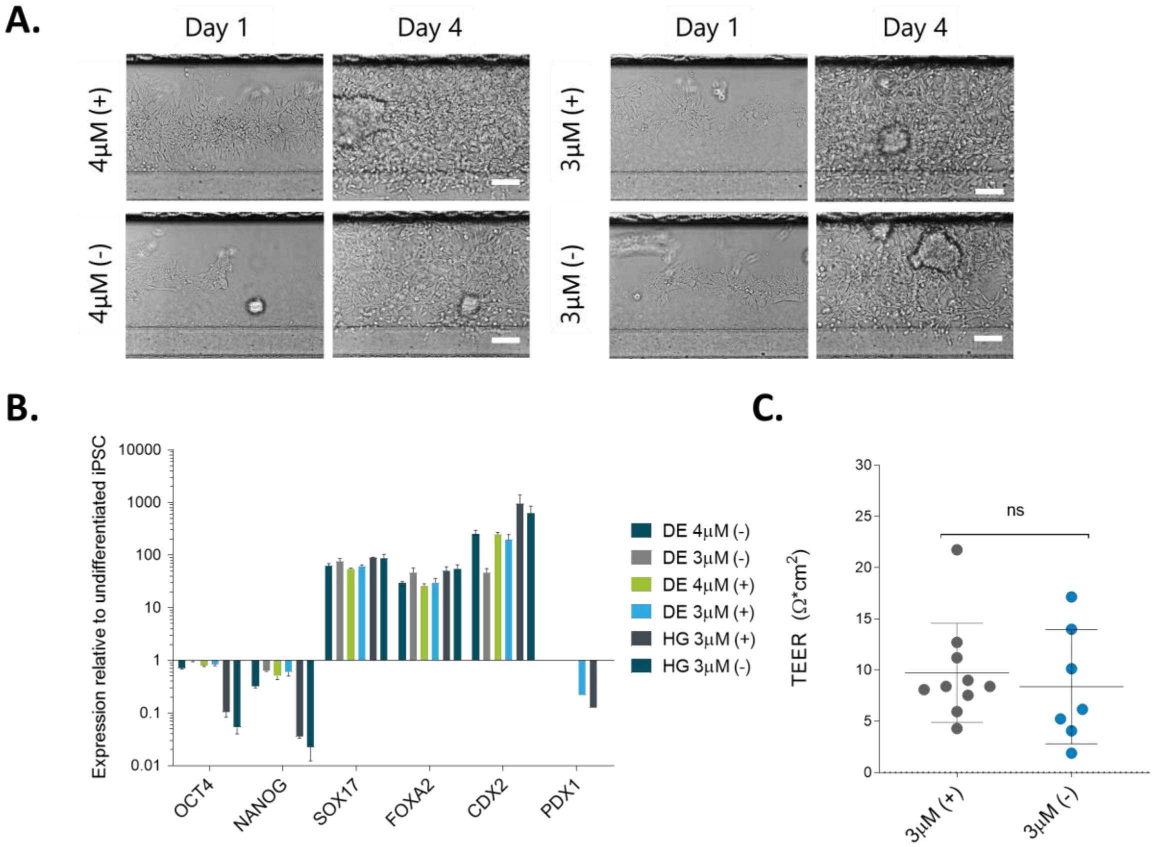

Figure S2. Endodermal potential screen and barrier integrity of iPSC. **A**. Representative phase contrast images of iPSC derived tubules at Day 1 and 4 cultured under four different conditions 3 or 4 µM CHIR99021 in RPMI supplemented with either B27 +/− insulin. Scale bars=100µm **B**. Gene expression were measured using TaqMan pRT-PCR at Day 4 (DE) for all four conditions and Day 7 (HG) for the 3µM CHIR99021 in RPMI supplemented with either B27 +/− insulin. The following genes were analyzed: Pluripotency: POU class 5 homeobox 1 (POU5F1); Nanog homeobox (NANOG) Primitive Streak:, forkhead box a2 (FOXA2) and Definitive Endoderm:FOXA2, SRY (sex determining region Y)-box 17 (SOX17) and markers for Anterior Gut: pancreatic and duodenal homeobox 1 (PDX1) and Posterior Gut Homeobox protein CDX-2. The Y-axis represents the LOG10 relative quantification (RQ). All samples were normalized to beta-actin (ACTB), and to undifferentiated hiPSC. Data is presented as the average of two independent experiments +/− SD (N=2, n≥3). **C**. TEER measurements of Day 4 hiPSC derived tubules at DE stage. Significance was tested by ordinary one-way Anova. Data is represented as mean ± SD . (N=1, n≥6), ns $p > 0.05$, * $p \leq 0.05$, ** $p \leq 0.01$, *** $p \leq 0.001$, **** $p \leq 0.0001$.

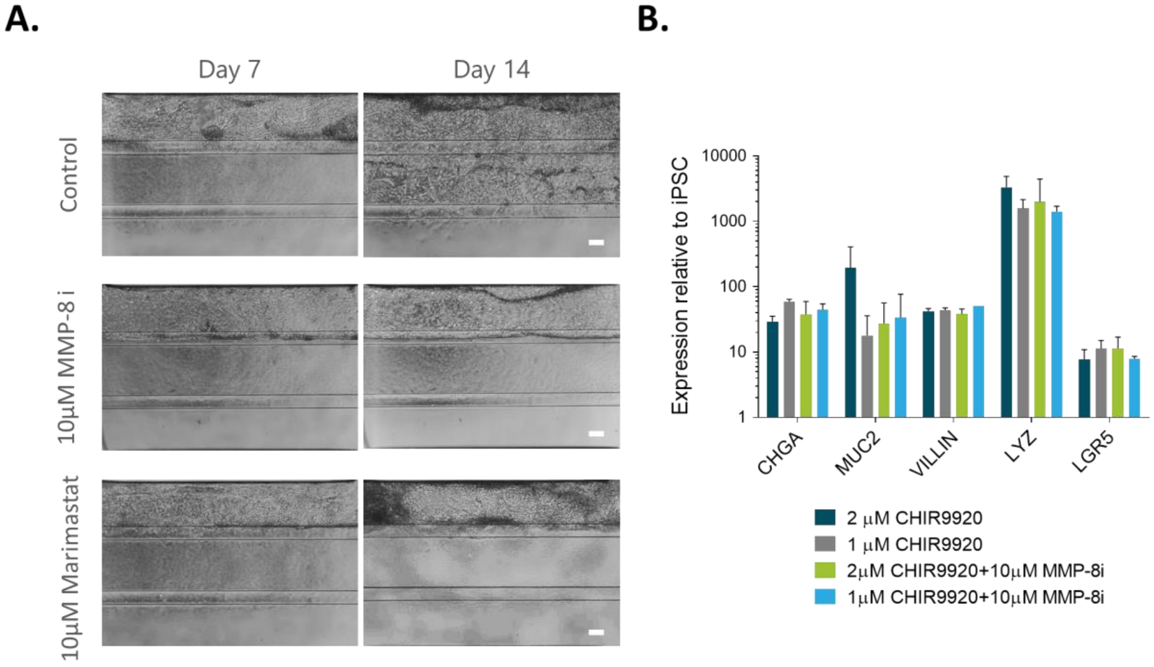

Figure S3. Maintenance of tubular shape of differentiated iPSC derived gut-like tubules within a microfluidic device with matrix metalloproteinases inhibitors. A. Representative phase contrast images on day 7 and day 14 per condition. Scale bars=100µm B. Gene expression measured using TaqMan qRT-PCR at day 28 from hiPSC derived gut-like tubules. The following genes were analyzed Leucine-rich repeat-containing G-protein coupled receptor 5 (LGR5), Mucin-2 (MUC2), Lysozyme (LYZ), Villin-1 (VIL1) and Chromogranin A (CHGA) The Y-axis represents the LOG10 relative quantification (RQ). All samples were normalized to beta-actin (ACTB) and expressed as relative to undifferentiated hiPSC. Data is presented as the average of two independent experiments +/− SD (n≥3).
